# Therapeutic Potential of Interferon-γ and Its Antagonists in Autoinflammation: Lessons from Murine Models of Systemic Juvenile Idiopathic Arthritis and Macrophage Activation Syndrome

**DOI:** 10.3390/ph8040793

**Published:** 2015-11-25

**Authors:** Anneleen Avau, Patrick Matthys

**Affiliations:** Laboratory of Immunobiology, Department of Microbiology and Immunology, Rega Institute, KU Leuven - University of Leuven, Leuven B-3000, Belgium; E-Mail: anneleen.avau@rega.kuleuven.be

**Keywords:** interferon-γ, therapy, autoinflammation, systemic juvenile idiopathic arthritis, mouse model

## Abstract

Interferon-γ (IFN-γ) affects immune responses in a complex fashion. Its immunostimulatory actions, such as macrophage activation and induction of T helper 1-type responsiveness, are widely acknowledged, however, as documented by a large body of literature, IFN-γ has also the potential to temper inflammatory processes via other pathways. In autoimmune and autoinflammatory disorders, IFN-γ can either play a disease-enforcing role or act as protective agent, depending on the nature of the disease. In animal models of any particular autoimmune disease, certain changes in the induction procedure can reverse the net outcome of introduction or ablation of IFN-γ. Here, we review the role of endogenous IFN-γ in inflammatory disorders and related murine models, with a focus on systemic juvenile idiopathic arthritis (sJIA) and macrophage activation syndrome (MAS). In particular, we discuss our recent findings in a mouse model of sJIA, in which endogenous IFN-γ acts as a regulatory agent, and compare with results from mouse models of MAS. Also, we elaborate on the complexity in the activity of IFN-γ and the resulting difficulty of predicting its value or that of its antagonists as treatment option.

## 1. Introduction

The family of interferons (IFNs) represents cytokines with viral “interference” properties. Three groups of IFNs have been described, based on their respective receptors. The type I IFN family is the largest, with IFN-α and IFN-β as archetypal members. All type I IFNs are related by structure and bind to a common heterodimeric receptor (IFNAR) [1]. The recently defined third subgroup of IFNs, consisting of IFN-λ subtypes, exerts actions similar to those of type I IFNs, but uses a different cell surface receptor (IFNLR). Interferon-γ (IFN-γ), discovered in the early 1960s, is the sole type II IFN. While IFN-γ owes its name and classification as an IFN to its antiviral activity [2], it differs from other IFNs in many ways [3]. Indeed, IFN-γ is encoded by a separate chromosomal locus, has its own receptor (IFNGR) and differs structurally from other types [4]. While both type I and type III IFNs are essential for antiviral immunity, the antiviral properties of IFN-γ represent only a subordinate part of its immunomodulatory functions. As deducted from studies in mice and individuals with errors in the IFN-γ pathways, a unique function of IFN-γ consists of defense against intracellular bacteria, fungi and parasites [5]. In contrast to type I IFNs, which are produced by virus-infected cells, IFN-γ is a product of immune cells in response to various stimuli. Predominant sources are natural killer (NK) cells and activated T cells, but B cells, NKT cells and antigen-presenting cells have also been described. Production and secretion of IFN-γ are under positive regulatory control by interleukin (IL)-12 and IL-18, cytokines that themselves are derived from antigen-presenting cells. IFN-γ orchestrates its many cellular reactions through induction of gene transcription via the Janus kinase (JAK)/signal transducer and activator of transcription (STAT)-pathway [4]. The role of IFN-γ in a certain disorder can be proinflammatory or regulatory, which explains the difficulty to predict its value (or that of its antagonists) as a treatment option.

## 2. Pro- and Anti-Inflammatory Aspects of Interferon-γ

IFN-γ both promotes proinflammatory pathways, like host defense against intracellular pathogens, and exerts regulatory functions, such as induction of T cell apoptosis. The net effect of IFN-γ signaling depends on the context and time frame wherein the disease progresses [6,7,8]. Table 1 provides an overview of prominent immune-stimulatory and anti-inflammatory effects of IFN-γ.

**Table 1:** General properties of interferon-γ in immune pathways.

Proinflammatory	Anti-Inflammatory
Macrophage activation	Inhibition of Th2 and Th17 polarization
Upregulation of antigen presentation pathways	Induction of IDO
NK cell activation	Induction of T cell apoptosis
Induction of Th1 polarization	Inhibition of neutrophil specific chemokines
Stimulation of monocyte chemoattractants	Inhibition of tissue damage
Upregulation of cell adhesion molecules	Inhibition of osteoclastogenesis
B cell maturation	Induction of IL-18 binding protein

Abbreviations: IDO, indoleamine 2,3-dioxygenase; NK, natural killer; Th, T helper.

### 2.1. Macrophage Activation and Enhancement of Immune Pathways

IFN-γ favors innate immune inflammatory responses through specific pathways in macrophage activation. These pathways include direct effects through the induction of effector gene transcription, and indirect effects through the enhancement of macrophage responsiveness to inflammatory stimuli [9]. Thus, IFN-γ induces the expression of MHC I and II antigens, the IL-2 receptor, complement receptors and several cytokines in a direct way. It enhances cytotoxicity of macrophages against tumor cells and infectious agents through induction of reactive oxygen and nitrogen species [3]. Indirectly, IFN-γ augments the sensitivity of macrophages to inflammatory triggers by increasing the expression of Toll-like receptors (TLRs) and inducing transcription factors that are essential for expression of TLR-responsive genes. As a result, the TLR-induced expression of inflammatory mediators and immune effectors, e.g., cytokines and chemokines, is augmented after IFN-γ priming [9,10]. As already indicated, IFN-γ promotes antigen presentation through upregulation of MHC molecules, entailing the increased expression of many different genes which all contribute to the antigen presentation pathways. IFN-γ is particularly specialized in the regulation of class II expression by antigen-presenting cells [11]. By this mechanism, it provides a link between innate and adaptive immunity. With respect to MHC I antigen presentation, IFN-γ plays an important role in the formation of the immunoproteasome. Thus, IFN-γ induces replacement of the three catalytically active β subunits of the proteasome into inducible catalytic subunits that ensure optimal quality and quantity of the generated peptides for presentation on MHC class I molecules [12].

NK cells are important sources of IFN-γ, particularly when stimulated with IL-12 and IL-18 [13,14]. In addition, IFN-γ is an enhancer of cytotoxic NK cell activity [15,16,17,18]. Accordingly, NK cells are defective in IFN-γ-deficient mice [19,20]. Of note, IFN-γ is the most potent inducer of IL-18 binding protein, a significant inhibitor of IL-18 [21]. By this regulatory function, IFN-γ induces a negative feedback pathway in inflammatory processes, also affecting its own production by NK cells. In B cells, IFN-γ acts directly on the secretion of antibodies by regulating the Ig class switching from IgM to a downstream isotype [11].

### 2.2. Modulation of T Helper and Regulatory T Cell Responses

**Figure 1:**
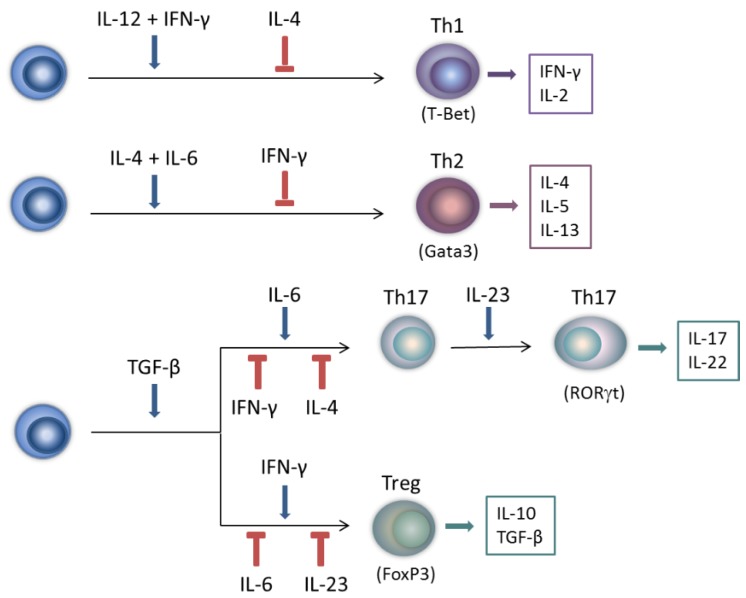
Polarization of T helper cell subsets and regulatory T cells. Naïve T cells (left) differentiate into different T helper (Th) lineages (Th1, Th2, and Th17) and regulatory T (Treg) cells. This polarization is stimulated or counteracted by different cytokine signals (indicated respectively by blue arrows and red lines). IL-12 and IFN-γ stimulate the development of Th1 cells, with T-Bet as master transcription factor. Th1 cells mainly produce IFN-γ and IL-2. IL-4 and IL-6 induce Th2 polarization. Th2 cells have Gata3 as major gene regulator, and predominantly produce IL-4, IL-5 and IL-13. TGF-β induces Th17 polarization when combined with IL-6, while IL-23 is required for Th17 differentiation. Signature products of Th17 cells are IL-17 and IL-22, while their master gene regulator is RORγt. In the absence of IL-6 or IL-23, TGF-β induces Treg cell formation, with FoxP3 as major transcription factor and IL-10 and TGF-β as major products. IFN-γ inhibits Th2 and Th17 cell polarization and stimulates Treg cell development under specific inflammatory conditions, while IL-4 suppresses Th1 and Th17 differentiation.

T helper (Th) cells are central players in the IFN-γ network cascades. They serve as important sources of IFN-γ, and their development is profoundly affected by the cytokine. Th cells are categorized depending on a set of polarizing cytokines, a particular cytokine production profile and relatively unique master gene regulators. Although commitment of Th cell lineages was originally seen as a unidirectional process of terminal differentiation, recent findings indicate that these processes are plastic and reversible, with important implications for autoimmune and autoinflammatory diseases [22]. IFN-γ and IL-2 are characteristic cytokine products of the Th1 population, while IL-4 is a marker of the Th2 lineage (Figure 1).

Differentiation towards Th1 cells is mainly induced by IL-12, but IFN-γ accelerates and enhances these effects. IL-4 and IL-6 are important inducers of Th2 cell formation. Thus, both IFN-γ and IL-4 auto-amplify responses of their producer T cell subgroup. Furthermore, IFN-γ and IL-4 are potent antagonists, cross-inhibiting differentiation and function of Th2 and Th1 cells [10,11]. In the early 2000s, an additional Th cell subgroup, the so-called Th17 population, was identified by the production of IL-17 [23]. Th17 cells were shown to be important for host defense against extracellular bacteria and yeasts, by promoting granulopoiesis, neutrophil infiltration and neutrophil activation. The Th17 subset is very heterogeneous and plastic. In contrast to Th1 and Th2 cells, which are induced by their own products, polarization of naïve T cells towards Th17 cells is not mediated by IL-17. IL-6 in combination with low concentrations of transforming growth factor (TGF)-β cooperate to induce Th17 cell differentiation, while IL-23 is needed downstream to stimulate proliferation and cytokine production [22,24]. Both IFN-γ and IL-4 inhibit polarization towards Th17 cells [24]. However, the relationship between Th17 and Th1 cells and between their signature products IL-17 and IFN-γ as opponents is complicated by the observation of IL-17/IFN-γ double-positive cells [25,26]. Elevated amounts of these cells can be detected in chronic inflammatory disorders, although their exact role in disease pathogenesis remains unclear. Finally, another T cell lineage, the regulatory T (Treg) cell subset, exerts important anti-inflammatory functions, e.g., restraining effector T cells, suppressing Th1, Th2 and Th17 cells, producing the anti-inflammatory IL-10 and maintaining homeostasis. Their development is induced by high concentrations of TGF-β, in the absence of proinflammatory cytokines such as IL-6 and IL-23. In certain inflammatory contexts, like experimental autoimmune encephalomyelitis (EAE) and collagen-induced arthritis (CIA), models for multiple sclerosis (MS) and rheumatoid arthritis (RA) respectively, IFN-γ favors regulatory over effector differentiation by stimulating Treg cell expansion [7]. As a consequence, it suppresses the formation of Th1 cells in an indirect negative feedback loop [10,22]. However, development of Treg cells proceeds normally in the absence of IFN-γ under several conditions [27]. Of note, IFN-γ indirectly promotes Treg development through the induction of indoleamine 2,3-dioxygenase (IDO), an immunomodulatory enzyme which catalyzes the degradation of tryptophan to kynurenine [28,29]. Furthermore, Koch *et al.* demonstrated that IFN-γ induced the expression of T-bet in FoxP3^+^ cells, representing a novel subset of Treg cells that dampens Th1 cell responses [30].

The mechanism by which IFN-γ regulates T cell immune circuits is not restricted to the modulation of Th cell commitment. Indeed, IFN-γ was demonstrated to be essential for the termination of immune responses by inducing apoptotic cell death in the T cell compartment in conditions of chronic inflammation with prolonged antigen stimulation and responses to self-antigens [31,32].

### 2.3. Influence on Chemokine Production and Attenuation of Tissue Destruction

Chemokines are produced by a variety of cells so as to attract specific immune cells. IFN-γ upregulates the expression of several chemokines, such as IFN-inducible protein (IP)-10 and monocyte chemoattractant protein (MCP)-1, as well as adhesion molecules, including intercellular adhesion molecule (ICAM)-1 and vascular cell adhesion molecule (VCAM)-1. These molecules specifically orchestrate the attraction of mononuclear cells to the inflammation spot [4]. Conversely, neutrophil attracting chemokine production is generally inhibited by IFN-γ [8].

Acute as well as chronic immune reactions cause collateral damage to the host, certainly when these reactions are not restrained in an appropriate way. IFN-γ precisely regulates the balance between clearance of pathogens and attenuation of inflammation-associated tissue damage. IFN-γ confines tissue damage by inhibiting the production of tissue-destructive factors, including matrix metalloproteinases, serine proteases and complement components [10]. Furthermore, tissue infiltration of neutrophils and monocytes is repressed by IFN-γ via several mechanisms: first, myelopoiesis and granulopoiesis are attenuated; secondly, neutrophil-attracting chemokine production is inhibited; and thirdly, cellular responsiveness to chemokines is altered by IFN-γ. Also, IFN-γ reduces damage in bone tissue as it suppresses formation of osteoclasts [7,8,10].

### 2.4. Recombinant IFN-γ as Therapy in Mycobacterial Infections

As indicated above, IFN-γ is indispensable in the immune defense against intracellular pathogens. Patients and mice with mutations in IFN-γ pathway genes are extremely susceptible for mycobacterial disease [5]. Therefore, recombinant IFN-γ is a conceivable treatment option for tuberculosis. Although previous clinical trials showed only moderate results, more studies are now conducted in different patient subgroups and with other routes of administration. Indeed, the results strongly depend on a combination of factors, including the dose, route and timing of drug administration, the immune status of the host and the type of infection [33].

## 3. Interferon-γ in Autoinflammation: Focus on Systemic Juvenile Idiopathic Arthritis

Autoinflammatory syndromes are characterized by episodes of seemingly unprovoked recurrent inflammatory attacks of an innate immune nature. In contrast to the traditionally defined autoimmune disorders, autoinflammatory diseases lack high-titer auto-antibodies or autoreactive T cells [34]. Systemic juvenile idiopathic arthritis (sJIA) is an example of such a disease, in which excessive activation of innate immune pathways dominates the inflammatory responses. A cytokine storm plays a signature role in the syndrome, as symptoms can be explained by the high levels of proinflammatory cytokines such as IL-6 and IL-18. Controversy exists, however, on the role of IFN-γ in sJIA [35]. In this section, we provide an overview of the pathogenesis of sJIA and its complication macrophage activation syndrome (MAS). We further elaborate on a recently developed CFA-induced mouse model of sJIA in which a protective role was assigned to IFN-γ and compare the model with findings from other CFA-induced experimental models as well as mouse models of MAS.

### 3.1. Pathogenesis of Systemic Juvenile Idiopathic Arthritis and Current Treatment Recommendations

Systemic juvenile idiopathic arthritis (sJIA), originally described by George Frederic Still in 1897 [36] and formerly known as systemic juvenile rheumatoid arthritis, systemic juvenile chronic arthritis, or Still’s disease, is a perplexing childhood disorder. The disease is classified as a subtype of juvenile idiopathic arthritis (JIA), a heterogeneous group of diseases defined by persistent arthritis of unknown origin that starts before the age of 16 and continues for at least 6 weeks [37]. JIA is the most common chronic rheumatic syndrome in childhood, with an incidence and prevalence that approximate to 10 and 100 cases per 100,000 children, respectively [38,39,40]. Based on the number of joints involved and the presence of extra-articular symptoms, the International League of Associations for Rheumatology (ILAR) classified JIA into seven different, mutually exclusive categories, of which oligoarticular and polyarticular JIA are the most common [37,38]. sJIA constitutes ~10% of all cases of JIA seen in North America and Europe, with a yearly incidence of ~0.8 cases per 100,000 children. Although sJIA can present at any time during childhood, a broad peak in the age of presentation was observed between 0 and 5 years, with 2 years of age most commonly reported [41]. Both sexes are equally affected [37,39,40]. Although classified as a subtype of JIA, symptoms are specific and different from those of other forms of childhood arthritis. According to the definition set by the ILAR [37], sJIA is characterized by arthritis in one or more joints, together with quotidian fever for at least 2 weeks, and accompanied by evanescent erythematous rash, generalized lymph node enlargement, hepatomegaly, splenomegaly and/or serositis. Exclusions from the sJIA diagnosis are made when other characteristic symptoms, like psoriasis or enthesitis, are encountered [37]. sJIA knows a highly variable presentation and course, with arthritis sometimes not developing until weeks to months after the first systemic signs. In general, systemic symptoms overshadow arthritis; the patients feel very ill and frequently develop weight loss [35,42,43]. It is therefore difficult to correctly diagnose sJIA: symptoms overlap with other conditions and the presentation differs substantially between patients. Although no specific laboratory tests exist for sJIA, the majority of patients has a typical pattern of systemic inflammatory markers. Characteristic abnormalities are high levels of C-reactive protein (CRP), high erythrocyte sedimentation rates (ESR), neutrophilia, thrombocytosis, and hypochromic normo- or microcytic anemia [42,43]. Autoantibodies are uncommon, but raised levels of polyclonal immunoglobins are repeatedly demonstrated. Elevated levels of ferritin and d-dimers are often seen, together with high levels of proinflammatory cytokines and chemokines [42,43]. Table 2 provides a detailed overview of generally described symptoms of sJIA.

The etiology of sJIA is largely unknown. Although infections are suspected as triggers, the absence of consistent seasonality and the low frequency in family members indicate that an infection is just one possible trigger among many others. One thought is that sJIA is initiated in children with a genetic susceptibility by infectious agents that are normally encountered during childhood. Once initiated, genetic polymorphisms contribute to the excessive reaction of the immune system. Associations with many genes have been described, supporting the idea that sJIA is multigenic and that different alleles are involved in the genetic predisposition [35,42,43,44]. In a first genomewide association study of sJIA, inherited risk factors were found within the class II human leukocyte antigen (HLA) locus [45,46]. Also, polymorphisms in non-HLA genes, e.g., in the promoter and genes encoding tumor necrosis factor (TNF)-α, the IL-1 family, IL-6 or IL-10, have repeatedly been described to be associated with sJIA [47,48,49,50,51]. Together, the associated polymorphisms in genes related to both innate and adaptive immunity demonstrate that sJIA is a complex disorder, suggesting contribution of both autoimmunity and autoinflammation. Of note, the presence of autoantibodies, autoreactive T cells or autoantigens has so far not been reported.

Though accounting for only 10% of the overall JIA prevalence, sJIA is responsible for most of the mortality seen in the disease group. An important cause of the high morbidity and mortality is its association with MAS, a life-threatening complication of several chronic childhood diseases which was first described in 1983 in association with sJIA [35,52]. MAS is characterized by excessive activation and/or expansion of T cells and macrophages, resulting in an overwhelming systemic inflammatory reaction and cytokine storm [53]. The overall clinical presentation of MAS includes an acute illness with unremitting high fever, hepatosplenomegaly, lymphadenopathy and mental status changes. Due to abnormalities in the coagulation profile, patients may have mucosal bleedings and purpura, resembling disseminated intravascular coagulopathy. Hallmark laboratory abnormalities are decreased blood cell numbers, *i.e.*, leukopenia, thrombocytopenia and anemia, highly elevated ferritin levels and elevated serum liver enzymes. A sudden fall in ESR despite persistently high CRP is also a distinctive feature. Triglycerides and lactate dehydrogenase levels are usually elevated, while sodium levels are depressed [53,54,55]. Symptoms of MAS overlap substantially with those of sJIA, making the recognition challenging. Several features, like lymphadenopathy and organomegaly, are common between the disorders. Other symptoms of MAS may be masked at first by the underlying sJIA symptoms. For example, blood values like platelet and neutrophil counts are increased during active sJIA, and may initially return to apparent normal levels during an MAS episode. As a result, changes only become evident at the late stage of the syndrome [53]. Table 2 provides an overview of MAS symptoms in a sJIA background. A pathognomonic feature of MAS is the appearance of hemophagocytic macrophages, *i.e.*, well-differentiated macrophages that engulf cells of hematopoietic origin [53]. Whether these macrophages are cause or consequence of the symptoms, and whether they exert a proinflammatory or rather a protective role in the disease, is still subject of investigations, but they serve as diagnostic markers [56]. The finding of prominent hemophagocytic activity by overactivated macrophages in patient bone marrow aspirates led to the term “macrophage activation syndrome” [57]. It is now widely acknowledged that the disease is a subtype of the hemophagocytic lymphohistiocytosis (HLH) syndromes [58], in which hemophagocytic macrophages are commonly demonstrated [59]. Due to the overlap in symptoms, sJIA and MAS are sometimes seen as the same entity within a spectrum of severity, with clinically unapparent MAS at one end and fulminant MAS at the other [58,60]. The demonstration of subclinical MAS symptoms, *i.e.*, hemophagocytosis in bone marrow aspirates of more than 50% of sJIA patients [61], certainly points in that direction.

**Table 2:** Symptoms of sJIA and MAS in association with sJIA [35,37,39,41,42,53,54,62,63,64,65,66,67].

	Feature	sJIA	MAS
	Incidence	~1/100,000	~10% of sJIA patients
**Clinical Features**	Fever	Quotidian	Persistent
	Rash	Evanescent	Petechial/macular
	Hepatomegaly	+	+
	Splenomegaly	+	+
	Lymphadenopathy	+	+
	Arthritis	+	-
	Serositis	+	-
	CNS dysfunction	−/+	+
**Laboratory Characteristics**	Neutrophil count	↑↑	↓
	Platelet count	↑↑	↓
	Anemia	+	+
	ESR	↑↑	Normal or ↓
	CRP	↑	↑
	ALT/AST	Normal or ↑	↑↑
	Fibrinogen	↑	↓
	Ferritin	Normal or ↑	↑↑
	D-dimers	↑	↑↑
	sCD25	Normal or ↑	↑↑
	sCD163	Normal or ↑	↑↑
**Histopathological Features**	NK cell dysfunction	Possible	Frequent
	Hemophagocytic macrophages	Possible	Frequent

+, often diagnosed; −/+, less commonly diagnosed/described; ↑↑, strong increase; ↑, increase; ↓, decrease. Abbreviations: ALT/AST, alanine aminotransferase/aspartate aminotransferase; CNS, central nervous system; CRP, C-reactive protein; ESR, erythrocyte sedimentation rate; NK, natural killer; sCD, soluble cluster of differentiation; sJIA, systemic juvenile idiopathic arthritis; MAS, macrophage activation syndrome in association with sJIA.

Both sJIA and MAS require a prompt diagnosis and management. In sJIA, therapy is started with nonsteroidal anti-inflammatory drugs in order to temper both joint pain and systemic features. In severe cases, corticosteroids are frequently prescribed, although long-term treatment with these drugs should be avoided to minimize side effects [42]. Traditionally, disease-modifying anti-rheumatic drugs, e.g., methotrexate and anti-TNF blockers, were used to minimize corticosteroid treatments. These therapeutics have proven their efficacy in other subtypes of JIA, but limited effectiveness was obtained in sJIA [43,44,68]. Since ongoing research in sJIA patients supports a disease-promoting role for IL-1β and IL-6 [69,70,71,72], a shift in treatment recommendations has been made towards IL-1 and IL-6 blockade. Both anti-IL-1β antibodies (canakinumab), IL-1 receptor antagonists (anakinra) and the anti-IL-6 receptor antibody tocilizumab were investigated in clinical trials, with promising results [73,74,75,76]. A subdivision of sJIA patients into subgroups was proposed based on the specific balance of IL-6 and IL-18, related to clinical profile, responsiveness to therapy or sensitivity to develop MAS [77]. The IL-6-dominant group presented mainly with arthritic symptoms, while the IL-18-dominant group seemed prone to develop MAS [78,79]. The subdivision based on the response to anakinra, as proposed by Gattorno and colleagues [80], can also be explained by an IL-1β/IL-18 dominant subgroup and an IL-6-dominant group [59,76]. The use of cytokine-related therapy is complex, as cytokine expression may arise in phases of disease and patients’ responses may alter during the disease course. In addition to cytokine-related therapy, other used therapeutics are cyclosporine A and thalidomide, both with moderate effectiveness and important side effects. Finally, autologous stem cell transplantation is considered in patients who have failed anti-IL-1 and anti-IL-6 treatment [42,43,81,82]. The importance of ongoing research into the pathogenesis of sJIA and the need for new treatment options are highlighted by the fact that a group of sJIA patients still fails to respond to recommended therapies or escapes after initial response [83].

### 3.2. IFN-γ in sJIA: Lessons from a New Mouse Model

The strong macrophage-activating potential of IFN-γ led to the consideration of the cytokine as proinflammatory agent in both sJIA and MAS. Indeed, IFN-γ levels are vastly increased in HLH patients [84,85,86,87,88], and murine models of the inherited form of HLH point to IFN-γ as the dominant causative cytokine [89,90]. In sJIA, the exact role of IFN-γ is subject of discussion. Despite the high inflammatory status of the patients, only moderately augmented IFN-γ levels have been detected in plasma. This observation is in sharp contrast with the highly elevated levels of IL-18, a signature IFN-γ-inducing cytokine [80,84]. Furthermore, de Jager and colleagues reported a defective IFN-γ production by NK cells of sJIA patients upon stimulation with IL-18 [91] and microarray analysis demonstrated an absence of IFN-γ -induced gene expression in peripheral blood mononuclear cells (PBMCs) from active sJIA patients [84,92,93]. When stimulating PBMCs of sJIA patients *in vitro* with IFN-γ, a normal response was noted [84,94]. All these data point to a limited exposure to IFN-γ *in vivo* in sJIA, arguing against a pivotal disease-enforcing role.

We recently described a new mouse model of systemic inflammation in IFN-γ-deficient BALB/c mice [20], in which symptoms correspond well to the characteristics of sJIA. The model relied on a single subcutaneous injection of complete Freund’s adjuvant (CFA), the most commonly used immunoadjuvant for the induction of experimental autoimmune diseases. CFA consists of a viscous emulsion of paraffin oil mixed with aqueous solutions or suspensions of antigens, and heat-killed mycobacteria (*Mycobacterium tuberculosis* or others). When antigens are emulsified in this oily adjuvant, their *in vivo* life span is prolonged resulting in an enhanced immune response. However, even in the absence of autoantigen both innate and adaptive immune reactions are provoked by CFA. The killed mycobacterial component acts as a booster for the immune response by eliciting extensive myelopoiesis and by skewing the immune response towards Th1 and γδ T cells [95,96,97]. The CFA-induced inflammation occurs in the first place at the site of injection and in the draining lymph nodes, where the hyperplasia, architectural changes and granuloma formation resemble a primary infection with living mycobacteria. Systemically, CFA acts through TLR activation, induction of cytokine and chemokine production and enhancement of antigen uptake by antigen-presenting cells. Hematopoietic remodeling takes place in the spleen, with disruption of the normal splenic architecture, leukocyte proliferation and granuloma formation [96]. In WT BALB/c mice, CFA challenge only results in a subtle and transient inflammatory reaction. IFN-γ-deficient mice however present with massive splenomegaly, lymphadenopathy, increased cytokine expression, anemia, thrombocytosis, granulocytosis, an immature blood cell profile and deficient NK cell cytotoxicity, all of which correspond to sJIA symptoms. Injection of CFA-treated WT mice with neutralizing antibodies against IFN-γ results in a comparable syndrome, indicating that a transient deficiency in IFN-γ suffices to induce sJIA-like symptoms (our own unpublished observations). Replacement of CFA by incomplete Freund’s adjuvant (IFA, not containing mycobacteria particles) has only subtle effects on the immune system [20], suggesting that the mycobacterial components in CFA account for the powerful immune reaction to the adjuvant, particularly in the absence of IFN-γ. As was mentioned earlier, IFN-γ (receptor)-deficient mice are extremely susceptible to infection with mycobacteria [98,99]. Therefore, the protective nature of IFN-γ in this context may result from the use of CFA and the consequent involvement of mycobacterium particles. However, the remarkable low levels of IFN-γ in sJIA patients, despite high IL-18 levels, are indicative of a protective function for the cytokine in the disease as well.

### 3.3. Comparison with CFA-Induced Experimental Autoimmune Diseases

In contrast to autoinflammatory disorders, autoimmune diseases, such as RA and MS, are characterized by immune reactions against self-antigens. IFN-γ-producing Th1 cells were historically heralded as signature pathogenic players in the onset and progress of these diseases. However, in associated experimental models, endogenous IFN-γ was found to fulfill a more subtle role, either augmenting or suppressing autoimmunity in a context-specific manner [7,100,101,102]. Evidence for the regulatory capacities of IFN-γ came from observations in CIA and EAE [7]. Like the sJIA-like model, those models rely on the use of CFA in the induction procedure. In both CIA and EAE, an aggravation of disease symptoms was noted in mice deficient in IFN-γ or its receptor [100,101,102]. It is now clear that both disease models are Th17-mediated, and that the protective effect of IFN-γ is most likely explained by its suppressive capacity on the differentiation and effector functions of the Th17 cells [22,103,104]. This assumption has been supported by the finding that ablation of IL-17, the major product of Th17 cells, counteracts the disease symptoms in mice with deficient IFN-γ pathways [105,106,107]. Of note, disease attenuation by IFN-γ in CIA and EAE is probably also mediated by other protective mechanisms, like suppression of neutrophil infiltration and tissue-destructive enzyme production, inhibition of osteoclastogenesis (CIA) and induction of Treg cell development [7,10].

It is an oversimplification to conclude that IFN-γ completely annihilates inflammation in RA and MS, as evident from results obtained in the corresponding CFA-induced mouse model. In CFA-independent models for RA, IFN-γ acts to enhance the inflammatory processes [98,108,109]. It is now commonly accepted that endogenous IFN-γ can exert both a disease-promoting and disease-mitigating role, depending on collateral conditions and on the time frame wherein the disease progresses [7,10,110]. Likewise, Th1 cells are not simply protective cell types in the diseases. A good understanding of the role of Th1 cells and IFN-γ in a specific autoimmune disease is advisable before considering therapeutic usage of antagonists of endogenous IFN-γ or of recombinant IFN-γ itself.

### 3.4. Comparison with Mouse Models of MAS

To compare the CFA-induced sJIA model with models independent of CFA, we highlight a mouse model covering the growth retardation seen in sJIA and two models with MAS-like features.

Chronic inflammation or recurrent infections in children have a vast effect on the normal growth rate, particularly during periods of augmented disease activity [111]. Stunted growth is a major complication of sJIA; the excessive production of IL-6 is one of the factors held responsible. The relation between high levels of IL-6 and reduced growth rates was demonstrated by De Benedetti *et al.* in neurospecific enolase (NSE)/hIL-6 mice, which have excessive expression of human IL-6 cDNA in mature neuron and neuroendocrine cells driven by the rat NSE promoter [112]. Growth defects were noted in some of the generated transgenic lines, with adult mice 50%–70% the size of WT littermates. Analysis of these lines demonstrated high circulating levels of IL-6, due to leaky expression in organs other than the central nervous system. Blockade of IL-6 restored the growth rate, indicating that the defects were a direct consequence of increased IL-6. Although no symptoms of arthritis were reported, the model could be of great help to increase our understanding of the aspect of growth retardation in sJIA and other chronic childhood disorders characterized by growth retardation [112,113]. Strippoli *et al.* developed a mouse model of MAS by administration of different TLR ligands to these IL-6 transgene mouse lines that displayed stunted growth [114]. LPS injection in the transgene mice resulted in increased lethality and elevated serum levels of IL-1β, IL-6, IL-18 and TNF-α compared to WT mice. Ferritin and sCD25 were increased, whereas platelet and neutrophil counts, and hemoglobin levels were significantly decreased. In vitro pretreatment of human macrophages with IL-6 likewise resulted in increased expression of cytokines when stimulated with LPS, indicating that exposure to high IL-6 levels affects the activation status of macrophages. This model provides insight into the development of MAS in sJIA. High IL-6 levels are a common feature of sJIA patients, which may partially explain the predisposition of sJIA patients to develop MAS after an infection. Concerning the role of IFN-γ in this model, it was recently described that treatment with antibodies against IFN-γ resulted in increased survival of the mice, assigning a proinflammatory role to the cytokine in this experimental system (abstract at the European Pediatric Rheumatology (PReS) Congress 2014) [115].

A second mouse model of MAS relies on chronic stimulation of TLR9 [116]. WT mice were given a series of intraperitoneal injections with CpG every two days for 10 days, resulting in pancytopenia, anemia, thrombocytopenia, splenomegaly, hepatitis, and hyperferritinemia. Serum levels of IL-12p70, IL-6, IL-10 and IFN-γ were elevated. TNF-α levels were not increased and blockade of the cytokine did not result in amelioration of the symptoms. Symptoms remained unchanged when only depleting NK cells or CD8^+^ cells, while mice lacking all lymphocyte populations showed attenuation of most of the disease parameters with exception of hepatitis. In IFN-γ-deficient mice, anemia, thrombocytopenia and hepatitis were absent, and splenomegaly was reduced, suggesting a proinflammatory role for IFN-γ. Leukopenia and hyperferritinemia were IFN-γ-independent. Hemophagocytosis, an important MAS symptom, was only observed after blocking IL-10, pointing to a protective function of IL-10 in the syndrome. In general, disruption of IL-10 resulted in a more severe disease, indicated by the authors as “fulminant MAS” [117]. Mice deficient in IFN-γ in which IL-10 was ablated developed essentially the same symptoms as WT mice when experiencing fulminant MAS, with exception of anemia. This model system indicates that MAS can be initiated by a hyperactive innate immune response. The MAS-like syndrome consists of a spectrum of disease severity depending on the levels of the protective IL-10. In the more severe disease conditions, *i.e.*, fulminant MAS, IFN-γ is not the causative agent, although increased levels were seen. Hemophagocytosis is associated with more severe disease, and may be part of a non-specific response to severe systemic inflammation, independent from the action of IFN-γ, but inhibited by IL-10.

Of note, both MAS models depend on the use of TLR triggers. The mouse model of Strippoli *et al.* indicates that chronic IL-6 exposure leads to an excessive amplification of immune responses after a single injection of TLR ligands [114]. In the CpG-induced model, MAS-like symptoms are provoked by repeated activation of TLRs [116].

### 3.5. Hypothesis

To summarize, the exact role of IFN-γ in sJIA remains complex. As for other experimental systems relying on CFA in the induction procedure, the CFA-induced sJIA-like syndrome indicates a protective role for IFN-γ in the disease. This regulatory function was not observed in MAS models, implying that IFN-γ may have a different role in sJIA *versus* MAS. However, a direct proinflammatory role of IFN-γ was also questioned in the CpG-dependent “fulminant MAS” model.

**Figure 2:**
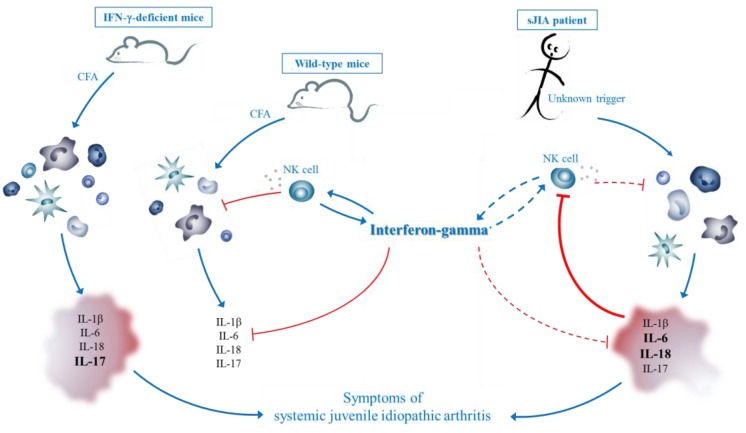
Proposed disease mechanism in mice and patients, with a central protective role of IFN-γ. (**Left**) In mice, complete Freund’s adjuvant (CFA) injection results in a local and systemic inflammatory reaction, leading to the expression of proinflammatory cytokines by activated immune cells. In wild-type mice (**middle**), IFN-γ counteracts the production of these cytokines by inhibiting IL-17-producing cells and by inducing NK cell cytotoxicity. Triggered NK cells kill activated immune cells by the release of cytotoxic granules. In the absence of IFN-γ (IFN-γ-deficient mice, **left**), NK cell function is defective, and activated immune cells keep producing proinflammatory cytokines. IFN-γ-associated inhibition of IL-17 is absent. As a consequence, the immune response is not ended properly, eventually resulting in typical sJIA-like symptoms; (**right**) In patients, an unknown trigger and genetic predisposition result in an excessive immune response, with high levels of proinflammatory cytokines, such as IL-6 and IL-18. These cytokines inhibit a proper NK cell functionality. As a consequence, less IFN-γ is produced and activated cells cannot be killed, resulting in the typical sJIA-like symptoms. Dashed arrows indicate impaired pathways.

In the sJIA model, we hypothesize that the anti-inflammatory features of IFN-γ are needed to appease chronic inflammation elicited by CFA, by inhibiting IL-17-producing cells and stimulating NK cell cytotoxicity and cytokine production (Figure 2, left and middle). In humans, an unknown trigger in genetically predisposed children starts a cascade of reactions with excessive production of proinflammatory cytokines, predominantly IL-6 and IL-18 (Figure 2, right). These cytokines may impair adequate functioning of NK cells. Indeed, it was recently demonstrated that high levels of IL-6 are associated with decreased NK cell function [118]. As a consequence, NK cells are unable to kill activated immune cells and terminate the immune response. Also, they fail to produce sufficient amounts of IFN-γ, which under normal circumstance may help to end inflammatory processes, e.g., via inhibition of IL-17-producing Th17 cells and through induction of IL-18 binding protein. Thus, in our opinion, the cause of the symptoms in the mouse model and humans may differ, but inadequate termination of the immune response eventually leads to similar symptoms, typically encountered in sJIA. IFN-γ may be essential to cooperate in an appropriate termination of inflammation under certain circumstances.

## 4. Therapeutic Potential of IFN-γ in Autoinflammatory Disorders

The pleiotropic effects of IFN-γ and its immunomodulatory functions imply a great therapeutic potential. The first clinical trials with recombinant human IFN-γ were conducted in 1986, defining its pharmacokinetics and side effects [119]. In more recently performed clinical trials, recombinant IFN-γ (actimmune or IFN-γ1b) and adenovirus vectors expressing IFN-γ cDNA have been used for treatment of a variety of disorders, including cancer, tuberculosis, hepatitis, osteopetrosis and scleroderma [119]. For the treatment of autoimmune diseases, both the use of recombinant IFN-γ and antibodies against IFN-γ (e.g., fontolizumab) have been considered, perfectly illustrating the dual role of the cytokine in these disorders [120,121,122,123,124].

In the inherited forms of HLH, a pathogenic role was assigned to IFN-γ based on different animal models [89,90], which has led to clinical trials with a new neutralizing antibody NI-0501. A first trial, investigating the safety of this new antibody in healthy volunteers, has been completed (ClinicalTrials.gov Identifier: NCT01459562). Clinicians are now recruiting patients with the inherited form of HLH for monitoring the safety and efficacy of NI-0501 and for long-term follow-up of patients treated with the drug (ClinicalTrials.gov Identifier: NCT01818492 and NCT02069899).

In sJIA, IFN-γ -related therapy is not under consideration at this time. Our results in mice certainly demonstrate that care should be taken with neutralization of IFN-γ, as they are suggestive of a protective role of the cytokine in the disease. In line with these assumptions, two research groups independently demonstrated effective treatment of sJIA with recombinant IFN-γ [125,126]. Mild side effects were observed, but the authors suggested a treatment strategy which involved exogenous IFN-γ in those sJIA patients that resisted to other treatments. Off course, the line between sJIA and MAS seems very thin, and either too little or too much IFN-γ could have devastating consequences. Therefore, a task that lies ahead for researchers is to deepen insights into the regulatory effects of IFN-γ in the diseases.

## 5. Concluding Remarks

While the role of IFN-γ in for example tuberculosis is clearly established, the exact contribution of the cytokine as proinflammatory or protective agent in autoinflammation remains a subject of dispute. The cytokine is involved in both pro- and anti-inflammatory circuits, thereby acting as a two-edged sword. Its proinflammatory properties, like macrophage activation and induction of Th1 polarization, serve the purpose of inducing a strong immune inflammatory reaction. On the other hand, important anti-inflammatory functions, including inhibition of T cell proliferation and induction of apoptosis, help to limit an excessive immune response in chronic inflammation. It may be important to note that many features of endogenous IFN-γ in immune processes have been established in mice in which IFN-γ was knocked out or neutralized by specific antibodies, and by using experimentally induced models of inflammation. Therefore some of the activities of IFN-γ may be related to the use of non-primates or may be conditioned by induction procedures (such as CFA). A better understanding of the biological activities of IFN-γ in human inflammatory disorders like sJIA and MAS is required, especially if the aim is to use this cytokine (or its antagonist) in medical practice. A detailed analysis of the disease, the patient and other treatment options may thus be necessary prior to considering IFN-γ-related therapy. Indeed, the administration of IFN-γ in an artificial way may result in side effects when dose or location do not correspond to the complex *in vivo* situation. A similar danger concerns anti-IFN-γ therapy, as blockade of the protective activities of the cytokine may have a great impact on chronic inflammation. The therapeutic window to use IFN-γ or its antagonists in autoinflammation may be narrow, depending on a combination of many factors.
